# Allergen immunotherapy for allergic rhinoconjunctivitis: protocol for a systematic review

**DOI:** 10.1186/s13601-016-0099-6

**Published:** 2016-03-22

**Authors:** Sangeeta Dhami, Ulugbek Nurmatov, Graham Roberts, Oliver Pfaar, Antonella Muraro, Ignacio J. Ansotegui, Moises Calderon, Cemal Cingi, Pascal Demoly, Stephen Durham, Ronald Gerth van Wijk, Susanne Halken, Eckard Hamelmann, Peter Hellings, Lars Jacobsen, Edward Knol, Desiree Larenas Linnemann, Sandra Lin, Vivian Maggina, Hanneke Oude-Elberink, Giovanni Pajno, Ruby Panwankar, Elideanna Pastorello, Constantinos Pitsios, Giuseppina Rotiroti, Frans Timmermans, Olympia Tsilochristou, Eva-Maria Varga, Jamie Wilkinson, Andrew Williams, Margitta Worm, Luo Zhang, Aziz Sheikh

**Affiliations:** Evidence-Based Health Care Ltd, Edinburgh, UK; Systematic Review at Decision Resources Group Abacus International, Bicester, UK; The David Hide Asthma and Allergy Research Centre, St Mary’s Hospital, Newport Isle of Wight, NIHR Respiratory Biomedical Research Unit, University Hospital Southampton NHS Foundation Trust, University of Southampton, Southampton, UK; Faculty of Medicine, University of Southampton, Southampton, UK; Department of Otorhinolaryngology, Head and Neck Surgery University Hospital, Mannheim and Center for Rhinology and Allergology, Wiesbaden, Germany; Food Allergy Referral Centre Veneto Region, Department of Women and Child Health, Padua General University Hospital, Padua, Italy; Hospital Quiron Bizkair, Bilbao, Spain; National Heart and Lung Institute, Imperial College, London, UK; Department of ENT, Eskisehir Osmangazi University Medical Faculty, Eskisehir, Turkey; University and Hospital of Montpellier, Inserm Paris Sorbonnes, Montpellier, France; Section of Allergology, Department of Internal Medicine, Erasmus MC, Rotterdam, The Netherlands; Hans Christian Andersen Children’s Hospital, Odense University Hospital, Odense, Denmark; Children’s Center Bethel, EvKB, Bieledelf and Allergy Center Buhr-University, Bochum, Germany; Laboratory of Experimental Immunology, University Hospitals Leuven, Louvain, Belgium; ALC, Allergy Learning and Consulting, Copenhagen, Denmark; University Medical Center, Utrecht, The Netherlands; Hospital Medica Sur, Mexico City, Mexico; Department of Otolaryngology-Head and Neck Surgery, John Hopkins, Baltimore, USA; Allergy and Clinical Immunology Unit, 2nd Department of Pediatrics, University of Athens, P and A Kiriakou Children’s Hospital, Athens, Greece; Department of Allergology, Groningen Research Institute for Asthma and COPD (GRIAC), University Medical Center Groningen, University of Groningen, Groningen, The Netherlands; Department of Pediatrics, University of Messina, Messina, Italy; Department of Pediatrics, Nippon Medical School, Tokyo, Japan; University of Milano, Milan, Italy; Department of Nutrition and Dietetics, Harokopio University, Athens, Greece; The Royal National Throat, Nose and Ear Hospital, University College London, London, UK; Netherlands Anafylaxis Network, Dordrecht, The Netherlands; Charite University Hospital, Berlin, Germany; Dept. of Paediatrics, Respiratory and Allergic Disease Division, Medical University Graz, Graz, Austria; Pharmaceutical Group of the European Union, Brussels, Belgium; Guy’s and St Thomas’ NHS Foundation Trust, London, UK; Chartie-Universitatsmedizin, Berlin, Germany; Beijing Institute of Otolarygology, Beijing, China; Allergy and Respiratory Research Group, The University of Edinburgh, Edinburgh, UK

**Keywords:** Allergy, Allergic rhinoconjunctivitis, Allergen immunotherapy, Rhinitis

## Abstract

**Background:**

The European Academy of Allergy and Clinical Immunology (EAACI) is in the process of developing the EAACI Guidelines for Allergen Immunotherapy (AIT) for the Management of Allergic Rhinoconjunctivitis. We seek to critically assess the effectiveness, cost-effectiveness and safety of AIT in the management of allergic rhinoconjunctivitis.

**Methods:**

We will undertake a systematic review, which will involve searching international biomedical databases for published, in progress and unpublished evidence. Studies will be independently screened against pre-defined eligibility criteria and critically appraised using established instruments. Data will be descriptively and, if possible and appropriate, quantitatively synthesised.

**Conclusion:**

The findings from this review will be used to inform the development of recommendations for EAACI’s Guidelines on AIT.

## Background

Allergic rhinoconjunctivitis is a very common chronic condition that can result in considerable morbidity and impairment of quality of life [1–3]. The disease is triggered by exposure to seasonal and/or perennial allergens and, depending on the nature of the allergenic trigger(s) and patterns of exposure, symptoms may be persistent or intermittent [4]. Allergic rhinitis is typically characterized by symptoms of nasal obstruction, a watery nasal discharge, sneezing and itching, and there is often (but not invariably) involvement of the conjunctiva, which manifests with itching, injection and tearing [5]. There may in addition be an impact on the ability to concentrate, on school and work performance, [6, 7] and interference with daily activities and sleep; furthermore, allergic rhinitis is a risk factor for the development of asthma [8].

Symptoms can, in many cases, be controlled with avoidance measures and conventional therapy such as oral, intranasal and intraocular H1-antihistamines, intranasal corticosteroids and anti-leukotrienes, as mono-therapy or in combination [4, 9, 10]. Allergen immunotherapy (AIT) is an additional treatment option, particularly for those with more troublesome disease which remains inadequately controlled by avoidance and pharmacotherapy [11–13]. The problem of uncontrolled rhinitis, despite treatment, continues to represent a therapeutic challenge in some patients [14].

The European Academy of Allergy and Clinical Immunology (EAACI) is in the process of developing the EAACI Guidelines for AIT, and this systematic review is one of five inter-linked evidence syntheses that are being undertaken in order to provide a state-of-the-art synopsis of the current evidence base in relation to evaluating AIT for the treatment of allergic rhinoconjunctivitis, food allergy, venom allergy and allergic asthma, and allergy prevention, which will be used to inform the formulation of key clinical recommendations. This review will focus on the effectiveness of AIT using key patient relevant outcomes: symptom and/or medication scores and disease specific quality of life [15]. It will also examine the cost-effectiveness and safety of AIT in the management of allergic rhinoconjunctivitis.

## Methods

### Search strategy

**Fig. 1:**
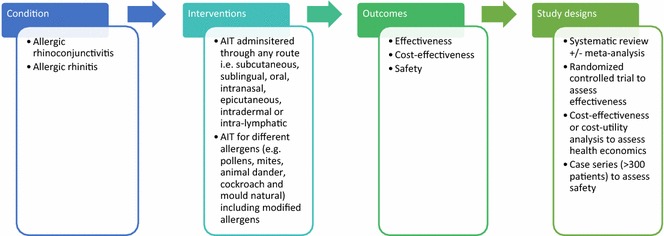
Conceptualization of systematic review of allergen immunotherapy for allergic rhinoconjunctivitis

A highly sensitive search strategy has been developed, and validated study design filters will be applied to retrieve articles pertaining to the use of AIT for allergic rhinoconjunctivitis from electronic bibliographic databases. We have conceptualized the search to incorporate the four elements shown in Fig. 1.

To retrieve systematic reviews, we will use the systematic review filter developed at McMaster University Health Information Research Unit (HIRU) (http://www.hiru.mcmaster.ca/hiru/HIRU_Hedges_MEDLINE_Strategies.aspx#Reviews). To retrieve randomized controlled trials (RCTs), we will apply the Cochrane highly sensitive search strategy for identifying RCTs in MEDLINE [16]. To retrieve case series, we will use the filter developed by librarians at Clinical Evidence: http://clinicalevidence.bmj.com/x/set/static/ebm/learn/665076.html.

We will search the following databases:

- Cochrane Library including,
  - Cochrane Database of Systematic Reviews (CDSR)
  - Database of Reviews of Effectiveness (DARE)
  - CENTRAL (Trials)
  - Methods Studies
  - Health Technology Assessments (HTA)
  - Economic Evaluations Database (EED)
- MEDLINE (OVID)
- Embase (OVID)
- CINAHL (Ebscohost)
- ISI Web of Science (Thomson Web of Knowledge)
- TRIP Database (http://www.tripdatabase.com)
- Clinicaltrials.gov (NIH web).
- Clinical trials register (http://www.clinicaltrialsregister.eu) launched by the European Medicines Agency (EMA)
- Current controlled trials (http://www.controlled-trials.com)
- Australian and New Zealand Clinical Trials Registry (http://www.anzctr.org.au).

The search strategy has been developed on OVID MEDLINE and then adapted for the other databases (see Appendix 1). In all cases, the databases will be searched from inception to October 31, 2015. Additional references will be located through searching the references cited by the identified studies, and unpublished work, while research in progress will be identified through discussion with experts in the field. We will invite experts who are active in the field from a range of disciplines and regions to add to the list of included studies by identifying additional published and unpublished papers they are aware of and research in progress. There will be no language restrictions employed; where possible, relevant literature will be translated into English.

### Inclusion criteria

#### Patient characteristics

We will focus on studies conducted on patients of any age with a physician-confirmed diagnosis of allergic rhinoconjunctivitis or allergic rhinitis, plus evidence of clinically relevant allergic sensitization (e.g., skin prick test or specific-IgE).

#### Interventions of interest and comparator

This review is focused on AIT for different allergens (e.g. pollens, house dust mites, animal dander, cockroach and moulds), including modified allergens, administered through any route [e.g. subcutaneous (SCIT), sublingual (SLIT), oral, intranasal, epicutaneous, intradermal or intra-lymphatic] compared with placebo or any active comparator.

#### Study designs

Systematic reviews of RCTs and RCTs will be used to investigate effectiveness, health economic analysis will be used to assess cost-effectiveness, and systematic reviews, RCTs and case series with a minimum of 300 patients will be used to assess safety (smaller case series are excluded in order to minimise selection biases).

#### Study outcomes

Primary

- Effectiveness, both short-term (i.e. during treatment) and long-term (i.e. at least a year after discontinuation of AIT) assessed by symptom and/or medication scores [16].

Secondary

- Assessment of disease specific quality of life
- Threshold of allergen exposure to trigger symptoms in an environmental exposure chamber or allergen challenge
- Safety as assessed by local and systemic reactions in accordance with the World Allergy Organization’s grading system of side-effects [17, 18]
- Health economic analysis from the perspective of the health system/payer.

### Exclusion criteria

The following exclusion criteria will be applied:

- Reviews, discussion papers, non-research letters and editorials
- Animal studies
- Quantitative studies not employing systematic review or RCT techniques
- Qualitative studies
- Case series (less than 300 patients).

### Study selection

All references will be uploaded into the systematic review software Distiller and undergo initial deduplication. Study titles will be independently checked by two reviewers according to the above selection criteria and categorized as: included, not included or unsure. For those papers in the unsure category, we will retrieve the abstract and re-categorize as above. Any discrepancies will be resolved through discussion and, if necessary, a third reviewer will be consulted. Full text copies of potentially relevant studies will be obtained and their eligibility for inclusion independently assessed. Studies that do not fulfil all of the inclusion criteria will be excluded.

### Quality assessment strategy

Quality assessments will independently be carried out on each study by two reviewers using the relevant version of the Critical Appraisal Skills Programme (CASP) quality assessment tool for systematic reviews and health economic evaluations [19, 20]. RCTs will be assessed for generation of allocation sequence, concealment of allocation, baseline outcome measurements, baseline characteristics, incomplete outcome data, blinding of outcome assessor, protection against contamination, selective outcome reporting and other risks of bias using the Cochrane Risk of Bias Tool. Similarly, we will use the quality assessment form produced by the National Institute for Health and Clinical Excellence (NICE) to critically appraise case series [21]. Any discrepancies will be resolved by discussion or, if agreement cannot be reached, a third reviewer will arbitrate.

### Data extraction, analysis and synthesis

Data will be independently extracted onto a customized data extraction sheet in Distiller by two reviewers, and any discrepancies will be resolved by discussion or, if agreement cannot be reached, by arbitration by a third reviewer.

A descriptive summary with summary data tables will be produced to summarize the literature. If clinically and statistically appropriate, meta-analysis using either fixed-effect or random-effects modeling will be undertaken [16]. A narrative synthesis of the data will also be undertaken. It is expected that it will only be appropriate to include the SLIT and SCIT studies in a meta-analysis to minimize heterogeneity; studies using other approaches will be described narratively.

### Sensitivity and subgroup analyses, and assessment for publication bias

Sensitivity analyses will be undertaken by comparing the summary estimates obtained by excluding studies considered to be at high risk of bias with those considered to be at low or moderate risk of bias.

Subgroup analyses will be undertaken to compare:

- Children (5–11) versus adolescents (12–17) versus adults (≥18 years)
- SCIT versus SLIT AIT
- Mild-to-moderate versus severe disease
- Aqueous solutions versus tablets in SLIT
- Modified allergen extracts (allergoids) versus unmodified allergen extracts in SCIT
- The use of single versus multiple allergens from different biological families in an extract
- AIT for seasonal versus perennial allergens
- Pre-seasonal (short term treatment) versus continuous treatment in SCIT
- Pre-/co-seasonal (short term treatment) versus continuous treatment in SLIT.

Where possible, publication bias will be assessed through the creation of funnel plots, and tested by Egger’s regression test and Begg’s rank correlation test [22, 23].

### Registration and reporting

This review will be registered with the International Prospective Register of Systematic Reviews (PROSPERO): http://www.crd.york.ac.uk/prospero/. The Preferred Reporting Items for Systematic Reviews and Meta-Analyses (PRISMA) checklist will be used to guide the reporting of the systematic review: http://www.prisma-statement.org/.

## Discussion

This review will involve systematically identifying, critiquing and synthesizing the evidence on the effectiveness, cost-effectiveness and safety of AIT for the management of allergic rhinoconjunctivitis. The review will take advantage of and build on other recent systematic reviews [24–27]. The findings from this review will be used to inform the development of recommendations for EAACI’s Guidelines on AIT. We anticipate that this review will report in 2016.

## Appendix 1: Search strategy

### Search strategy 1

#### (MEDLINE, EMBASE)

1. exp Rhinitis/
2. Rhinitis Allergic Perennial/
3. Rhinitis, allergic, seasonal/
4. hayfever.mp.
5. hay fever.mp.
6. fever, hay.mp.
7. seasonal allergic rhinitis.mp.
8. allergic rhinitides.mp.
9. allergic rhinitis.mp.
10. rhiniti*.mp.
11. pollinosis.mp.
12. pollenosis.mp.
13. exp Nasal obstruction/
14. Conjunctivitis/
15. Conjunctivitis, Allergic/
16. conjunctivit*.mp.
17. rhino-conjunctivit*.mp.
18. or/1-17
19. exp Desensitization, Immunologic/
20. exp Immunotherapy/
21. Desensitization.mp.
22. Immunotherapy.mp.
23. Oral Immunotherapy.mp.
24. Oral desensitization.mp.
25. Sublingual Immunotherapy.mp.
26. Subcutaneous Immunotherapy.mp.
27. Epicutaneous Immunotherapy.mp.
28. Intradermal Immunotherapy.mp.
29. (Intra-lymphatic or intra lymphatic immunotherapy).mp.
30. Intranasal Immunotherapy.mp.
31. Specific Immunotherapy.mp.
32. Or/19-31
33. exp Intervention Studies/
34. Intervention Studies.mp.
35. Experimental stud*.mp.
36. exp Clinical Trial/
37. Trial.mp.
38. Clinical Trial.mp.
39. exp Controlled Clinical Trial/
40. Controlled Clinical Trial.mp.
41. Randomi?ed Controlled Trial.mp.
42. exp Placebos/
43. Placebos.mp.
44. exp Random Allocation/
45. Random Allocation.mp.
46. exp Double-Blind Method/
47. Double-Blind Method.mp.
48. Double-Blind design.mp.
49. exp Single-Blind Method/
50. Single-Blind Method.mp.
51. Single-Blind design.mp.
52. Triple-Blind Method.mp.
53. Random*.mp.
54. Search:.tw
55. Review.pt.
56. Systematic review.tw.
57. Meta analysis.mp,pt.
58. Case series.mp.
59. (Case$ and series).tw.
60. Cost:.mp.
61. Cost effective:.mp
62. Exp Health Care Costs/
63. (Costs and Costs Analysis).mp.
64. Economic evaluation*.mp.
65. ((cost effective* adj1 analys*) or cost minimi?ation analys* or cost benefit analys* or cost utility analys* or cost consequence analys* or finances).mp.
66. Or/33-65
67. 18 and 32 and 66

### Search strategy 2

(Cochrane library, TRIP, CINAHL, ISI Web of Science, HTA, EED) (Rhinitis* or allergic rhinitis or allergic rhinitides or seasonal allergic rhinitis or hayfever or hay fever or poll?nosis or pollenosis or conjunctivit* or allergic conjunctivitis or rhino conjunctivitis or rhino-conjunctivitis) and (Immunologic, desensiti* or immunotherapy or oral immunotherapy or oral desensiti?ation or sublingual immunotherapy or subcutaneous immunotherapy or epicutaneous immunotherapy or intradermal immunotherapy or intra-lymphatic immunotherapy or intranasal immunotherapy) and (Intervention stud* or experimental stud* or trial or clinical trial* or controlled clinical trial or randomi* controlled trial or random allocation or single blind method or double blind method or triple blind method or random* or systematic review or meta-analysis or case series or economic evaluation* or cost effective* analys* or cost minimi?ation analys* or cost benefit analys* or cost utility analys* or cost consequence analys* or finances)
