# Erratum to: Allergen immunotherapy for allergic rhinoconjunctivitis: protocol for a systematic review

**DOI:** 10.1186/s13601-017-0168-5

**Published:** 2017-09-15

**Authors:** Sangeeta Dhami, Ulugbek Nurmatov, Graham Roberts, Oliver Pfaar, Antonella Muraro, Ignacio J. Ansotegui, Moises Calderon, Cemal Cingi, Pascal Demoly, Stephen Durham, Ronald Gerth van Wijk, Susanne Halken, Eckard Hamelmann, Peter Hellings, Lars Jacobsen, Edward Knol, Desiree Larenas Linnemann, Sandra Lin, Vivian Maggina, Hanneke Oude-Elberink, Giovanni Pajno, Ruby Panwankar, Elideanna Pastorello, Constantinos Pitsios, Giuseppina Rotiroti, Frans Timmermans, Olympia Tsilochristou, Eva-Maria Varga, Jamie Wilkinson, Andrew Williams, Margitta Worm, Luo Zhang, Aziz Sheikh

**Affiliations:** 1 Evidence-Based Health Care Ltd, Edinburgh, UK; 2 Systematic Review at Decision Resources Group Abacus International, Bicester, UK; 3 The David Hide Asthma and Allergy Research Centre, St Mary’s Hospital, Newport Isle of Wight, NIHR Respiratory Biomedical Research Unit, University Hospital Southampton NHS Foundation Trust, University of Southampton, Southampton, UK; 4 Faculty of Medicine, University of Southampton, Southampton, UK; 5 Department of Otorhinolaryngology, Head and Neck Surgery University Hospital, Mannheim and Center for Rhinology and Allergology, Wiesbaden, Germany; 6 Food Allergy Referral Centre Veneto Region, Department of Women and Child Health, Padua General University Hospital, Padua, Italy; 7 Hospital Quiron Bizkair, Bilbao, Spain; 8 National Heart and Lung Institute, Imperial College, London, UK; 9 Department of ENT, Eskisehir Osmangazi University Medical Faculty, Eskisehir, Turkey; 10 University and Hospital of Montpellier, Inserm Paris Sorbonnes, Montpellier, France; 11 Section of Allergology, Department of Internal Medicine, Erasmus MC, Rotterdam, The Netherlands; 12 Hans Christian Andersen Children’s Hospital, Odense University Hospital, Odense, Denmark; 13 Children’s Center Bethel, EvKB, Bieledelf and Allergy Center Buhr-University, Bochum, Germany; 14 Laboratory of Experimental Immunology, University Hospitals Leuven, Louvain, Belgium; 15 ALC, Allergy Learning and Consulting, Copenhagen, Denmark; 16 University Medical Center, Utrecht, The Netherlands; 17 Hospital Medica Sur, Mexico City, Mexico; 18 Department of Otolaryngology-Head and Neck Surgery, John Hopkins, Baltimore, USA; 19 Allergy and Clinical Immunology Unit, 2nd Department of Pediatrics, University of Athens, P and A Kiriakou Children’s Hospital, Athens, Greece; 20 Department of Allergology, Groningen Research Institute for Asthma and COPD (GRIAC), University Medical Center Groningen, University of Groningen, Groningen, The Netherlands; 21 Department of Pediatrics, University of Messina, Messina, Italy; 22 Department of Pediatrics, Nippon Medical School, Tokyo, Japan; 23 University of Milano, Milan, Italy; 24 Department of Nutrition and Dietetics, Harokopio University, Athens, Greece; 25 The Royal National Throat, Nose and Ear Hospital, University College London, London, UK; 26 Netherlands Anafylaxis Network, Dordrecht, The Netherlands; 27 Charite University Hospital, Berlin, Germany; 28 Dept. of Paediatrics, Respiratory and Allergic Disease Division, Medical University Graz, Graz, Austria; 29 Pharmaceutical Group of the European Union, Brussels, Belgium; 30 Guy’s and St Thomas’ NHS Foundation Trust, London, UK; 31 Chartie-Universitatsmedizin, Berlin, Germany; 32 Beijing Institute of Otolarygology, Beijing, China; 33 Allergy and Respiratory Research Group, The University of Edinburgh, Edinburgh, UK

## Erratum to: Clin Transl Allergy (2016) 6:12 DOI 10.1186/s13601-016-0099-6

Unfortunately this article [1] was published with an error in the Funding section. The BM4SIT project is not acknowledged. This section should be corrected to the below:

**Funding**

EAACI and the BM4SIT project (Grant Number 601763) in the European Union’s Seventh Framework Programme FP7.

## References

[1] Dhami S (2016). Allergen immunotherapy for allergic rhinoconjunctivitis: protocol for a systematic review. Clin Transl Allergy.

